# Maintaining Klotho: preventing the body from unwinding

**DOI:** 10.3389/fragi.2026.1913194

**Published:** 2026-10-27

**Authors:** Walter H. Gunzburg, Pharon Pawa, Brian Salmons

**Affiliations:** 1 Department of Biological Sciences and Pathobiology, University of Veterinary Medicine, Vienna, Vienna, Austria; 2 Austrianova Thailand, Thailand Science Park, Khlong Luang, Pathumthani, Thailand

**Keywords:** aging, cell therapy, Cell-in-a-Box, encapsulation, gene therapy, Klotho, longevity, regenerative medicine

## Abstract

The Klotho gene, discovered in 1997, encodes a protein that plays a pivotal role in regulating aging and longevity. Reduced Klotho expression is associated with multiple age-related diseases, including renal, cardiovascular, neurodegenerative, and metabolic disorders, leading to the notion that supplementation of the levels of Klotho may have a beneficial, or even a therapeutic, effect. Despite promising early stage results from recombinant and gene-based therapies, clinical translation has been hindered in part by the protein’s short half-life as well as the immune clearance of Klotho producing cells. This article reviews Klotho’s biological significance and therapeutic challenges, as well as introducing a novel encapsulation-based approach—Cell-in-a-Box® technology—as a proposed potential solution for sustained Klotho delivery. Encapsulated Klotho-producing cells may potentially enable continuous secretion, immune protection, and long-term efficacy, offering a transformative strategy for anti-aging and disease modification.

## 1 Introduction

Since its discovery by Kuro-o and colleagues in 1997 (Kuro-o et al., 1997), the Klotho gene has been recognized as a master regulator of aging and longevity. Named after the mythological spinner of life’s thread (Nambidi et al., 2025), Klotho symbolizes the maintenance of physiological balance and integrity—a guardian of the body’s molecular weave. This has been demonstrated directly, i.e., when the Klotho gene is impaired, inactivated or “knocked out” in mice, the resulting animals show a specific syndrome that strongly resembles premature ageing–not only is the lifespan of the mice shortened, but they also show ectopic calcification and increased arteriosclerosis and osteoporosis (Kuro-o et al., 1997). In contrast, over-expression of Klotho in mice has been shown to inhibit the ageing process resulting in an increased life span by 20%–30% (Kurosu et al., 2005). This remarkable contrast underscores Klotho’s central role in preserving cellular and systemic health.

As Klotho levels decline with age, the body’s intricate equilibrium begins to unravel, leading to progressive deterioration across multiple organ systems (Nambidi et al., 2025). Restoring its expression has therefore become a compelling therapeutic goal, though achieving sustained production remains challenging due to the protein’s rapid degradation and transient expression in conventional delivery systems. The metaphor of “mending old clothes” aptly captures Klotho’s biological function—repairing metabolic wear, reinforcing cellular resilience, and maintaining the weave of life itself.

## 2 Biological role of Klotho (sKL)

Klotho exists in two principal forms: a membrane-bound co-receptor for fibroblast growth factor 23 (FGF23) and a soluble endocrine factor (Kuro-o, 2011). Together, these forms orchestrate mineral metabolism by regulating phosphate and calcium balance, while also exerting broader systemic effects (Nambidi et al., 2025). Soluble Klotho (sKL), in particular, has been shown to modulate insulin sensitivity, reduce oxidative stress, and counteract fibrotic and inflammatory processes (Zhu et al., 2025), positioning it as a multifaceted guardian of physiological integrity (see Figure 1).

**FIGURE 1:**
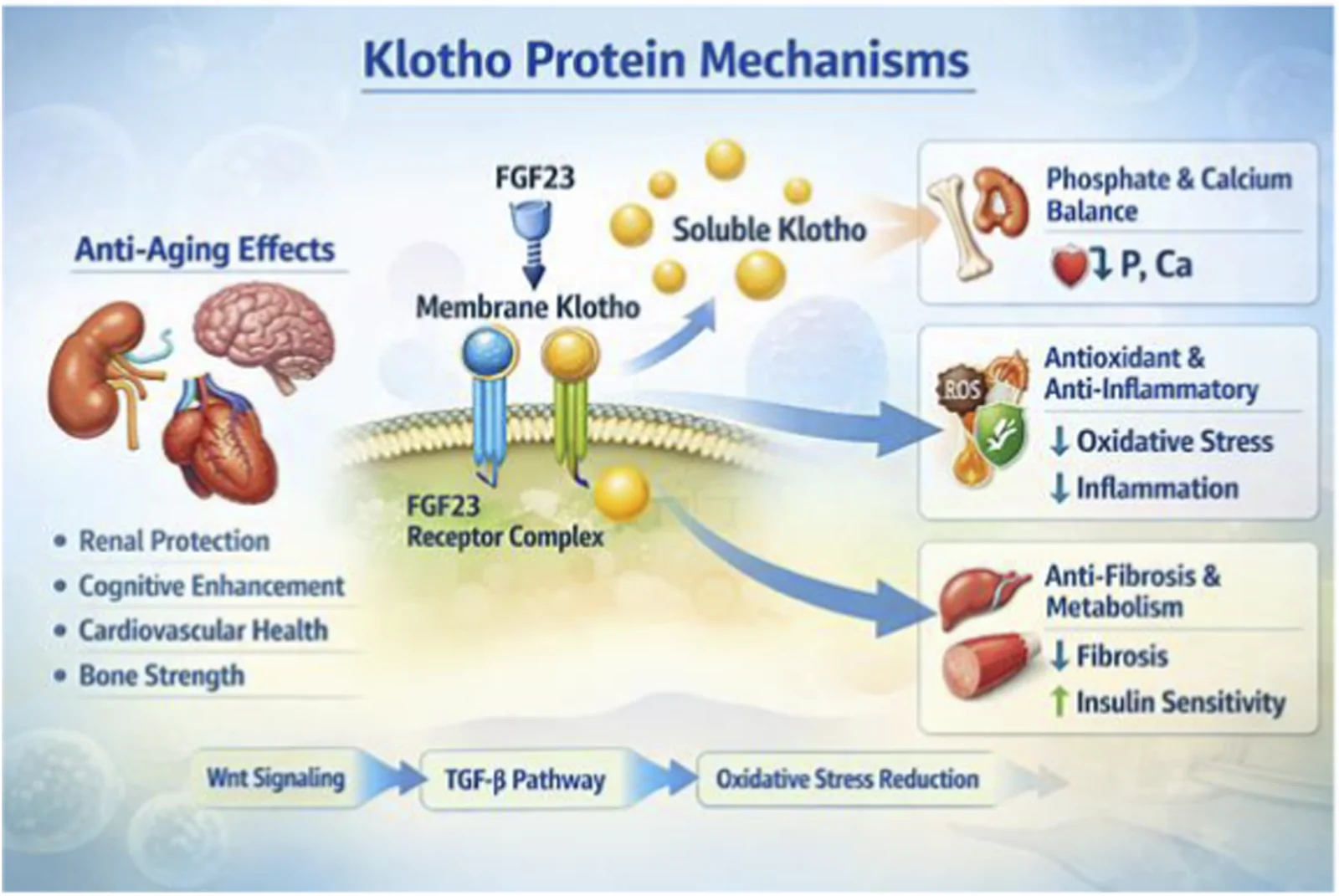
Known mechanisms of Klotho protein action. Illustrates membrane-bound and soluble Klotho forms. The membrane form acts as an FGF23 co-receptor regulating phosphate and calcium balance, while soluble Klotho exerts systemic anti-oxidative, anti-inflammatory, and anti-fibrotic effects. Arrows show downstream signaling through Wnt and TGF-β pathways contributing to metabolic homeostasis.

Excitement about this protein has surged over the years, not only because of its association with general anti-aging properties (Roig-Soriano et al., 2025), but also with the subsequent finding that diminished Klotho levels correlate with a whole series of diseases and conditions that generally arise in later life. This has lead to the hypothesis that when Klotho levels decline, the consequences ripple across multiple organ systems. In the kidneys, reduced expression is linked to chronic and diabetic kidney disease, acute injury, and progressive fibrosis (Zununi Vahed et al., 2013). In the cardiovascular system, deficiency contributes to arteriosclerosis, vascular calcification, and hypertension (Ding and Ma, 2015). Indeed, failing cardiovascular health drives down Klotho levels, which accelerates vascular aging, calcification, and heart failure. Conversely, prioritizing cardiovascular health sustains endogenous Klotho expression, utilizing the body’s native anti-aging pathways to protect the heart against age-related decline (Hellou et al., 2025). Neurodegenerative conditions such as Alzheimer’s, Parkinson’s (López-Valdés et al., 2025), and amyotrophic lateral sclerosis (Zeldich et al., 2019) have all been associated with diminished Klotho activity, while metabolic decline manifests sarcopenia, osteoporosis (Ariadel-Cobo et al., 2025), and even chronic obstructive pulmonary disease (COPD) (Shi et al., 2024). Cancer biology also reflects this vulnerability, with Klotho acting as a tumor suppressor - reduced Klotho expression correlating with tumor progression in pancreatic, breast, and oral carcinomas (Prud’homme and Wang, 2026).

The breadth of these associations underscores the notion that Klotho may have a central role in maintaining systemic resilience. If this notion is true, then the restoration of Klotho levels to those found in childhood and youth may potentially offer the tantalizing possibility of simultaneously mitigating diverse degenerative pathways, making Klotho not only a biomarker of aging but also a potential unifying therapeutic target in longevity research.

## 3 Therapeutic challenges

Despite its extraordinary potential as a possible therapeutic agent, Klotho therapy however continues to face formidable obstacles. One is the very short half-life of the protein, which has been measured as around 7.5 h in human serum (Hu et al., 2016), making the maintenance of therapeutic levels difficult. Further, studies have shown that circulating Klotho is also rapidly cleared by the kidneys (Hu et al., 2016). Another potential challenge is that there is currently no established human standard or FDA-approved dosage for Klotho protein or peptide treatments. The pharmacological development of Klotho requires precise dosing that balances its therapeutic potential against potential risks and consequences of systemic disruption. Indeed, because Klotho acts as a coreceptor for fibroblast growth factor 23 and regulates major metabolic, inflammatory, and ion pathways, managing its exposure is a critical aspect of clinical translation as is long-term safety monitoring (for a comprehensive review see (Zeng et al., 2026).

Gene therapy seems like one obvious way to genetically modify the patient’s own cells to produce Klotho, and indeed preclinical results using adeno associated virus vectors (e.g., AAV9) to deliver Klotho protein to the central nervous system could provide a potential strategy to treat Alzheimer’s and ALS (Massó et al., 2018). In July 2025, Klotho Neurosciences (now Greenland Mines) received Orphan Drug Approval from the US-FDA for their myotropic adeno-associated virus (AAVMYO2) gene therapy vector containing sKL and a muscle-specific promoter for the treatment of amyotrophic lateral sclerosis. The candidate therapeutic, called KLTO-202, is being evaluated in a Phase 1 trial at UCSF that opened in early 2025 and is now actively dosing in 2026. The trial has a single ascending dose study design in healthy older adults and aims to evaluate safety, pharmacokinetics, and a panel of cognitive endpoints over a 90 days window after a single subcutaneous injection (Healthcare Discovery, 2026). Nevertheless, AAV vectors are known from other studies to trigger immune responses, and this also limits the number of times these vectors can be administrated to patients (Yi et al., 2025).

The company Minicircle has just completed a phase 1 trial in healthy human volunteers (Minicircle, 2026) in Próspera, Honduras. The aim of the trial was to investigate the safety and efficacy of a combination klotho and follistatin gene therapy, delivered *via* a subcutaneous fat injection of plasmid DNA and transformation agent designed to facilitate uptake of the DNA into the patient’s genome, as well as to understand the cognitive and health benefits of this gene therapy (Minicircle, 2026). Similarly, the promising results obtained from delivery of klotho plasmid DNA complexed with nano-particles in animals (Li et al., 2025) suggests that nanoparticles may be a possible alternative to pure plasmid DNA transfection in clinical trials. Unfortunately, although early studies in animal models suggested long term expression with durations in muscle of over 19 months (Wolff et al., 1992), more recent studies have reported much shorter transgene expression or detection in human clinical trials, often only lasting 1 month (Gothelf et al., 2010).

Lipid Nanoparticle (LNP) delivery systems are also being tested as a means to deliver the Klotho mRNA in clinical trials, such as those initiated by Klothea Bio. Their randomized, double-blind, placebo-controlled Phase 1b Clinical Trial of AKL003 at the GARM Clinic in Roatan, Honduras, is designed to increase human lifespan (Klothea Bio, 2026). While this a promising approach, unfortunately, LNPs face issues of potential toxicity, low delivery efficiency to tissues outside the liver (80%–90% of systemically administered LNPs accumulate in the liver, leaving less than 5% for the intended target tissues), as well as being easily recognized and cleared by the mononuclear phagocytic system (Wang et al., 2024).

Thus, conventional delivery systems such as adeno-associated viral vectors, plasmid constructs, or lipid nanoparticles can induce transient gene expression, but the benefits fade as expression is lost.

Cell therapy where stem cells or genetically modified cells producing Klotho are transplanted into the patient’s body seems also to be an obvious treatment strategy (Elshari, 2025). However, Klotho produced from cells (at least in cell culture) can have an even shorter half-life (Tucker Zhou et al., 2013). Also, such implanted cells are often rapidly lost from the body either through elimination by the immune system within days (Ankrum et al., 2014) and/or through physical clearance of the cells (Collins et al., 2017; Collins et al., 2017), limiting their durability so that Klotho production is again lost.

So whilst cell and gene therapy strategies and trials, including those using AAV9 vectors for neurodegenerative diseases such as amyotrophic lateral sclerosis (ALS) and Alzheimer’s, have demonstrated potential, they remain constrained by immune activation and the instability of long-term expression. These challenges highlight the need for innovative strategies capable of sustaining Klotho delivery over time, ensuring that its protective effects can be harnessed without being undermined by biological barriers.

## 4 Encapsulation technology: a durable solution

Several technologies have been explored for sustained *in vivo* delivery of therapeutic proteins, including biodegradable protein depots, implantable infusion devices, and cell-encapsulation systems. While each approach offers distinct advantages, significant differences exist in their suitability for chronic delivery of a labile protein such as Klotho.

Mechanical delivery systems, including mini-osmotic pumps, provide highly controlled dosing and predictable pharmacokinetics but are fundamentally limited by finite reservoir capacity, the need for periodic replacement or refilling, and potential instability of proteins during prolonged storage within the device (Hu et al., 2017). Lionheart Health is developing an implantable “Octopus” pump system to deliver Klotho protein in a controlled, repeatable manner. This is an implantable pump with a reservoir which can be filled percutaneously with soluble Klotho protein. Further, as part of its KlothoPatch™ II bioelectronic organ bioreactor platform, the reservoir can contain a nutrient hydrogel which can be used in conjunction with Klotho-overexpressing mesenchymal stem cells (MSCs) to provide long term Klotho production, essentially combining the pump mechanical system with a cell-encapsulation system (Lionheartlongevity, 2026).

Hydrogels and advanced biomaterial depots have also been used for Klotho protein delivery in animal models. PLA and PLGA microparticles represent clinically established platforms capable of controlled protein release through tunable polymer degradation; however, these systems rely on a fixed protein payload, cannot accommodate living cells, provide no immunoprotection, and may expose encapsulated proteins to acidic degradation products that can compromise biological activity (Jumadi et al., 2026). PLGA scaffolding however has been used to provide structural integrity to photocrosslinked silk fibroin methacryloyl (SilMA) -based hydrogels containing both Klotho protein and MSCs. Such bio-active PLGA/SilMA hydrogels were able to enhance both structural and functional regeneration of myotendinous junctions in a rat model (Sun et al., 2024). A similar system based on chitosan hydrogels has also been successfully shown to allow long term Klotho release in murine models of renal fibrosis (Li et al., 2024).

In contrast, cell-encapsulation technologies offer the unique advantage of continuous *in situ* production of therapeutic proteins by viable implanted cells, potentially eliminating the need for repeated administration. Alginate microcapsules are the most extensively studied encapsulation system and have demonstrated utility in a wide range of cell therapy applications; however, their long-term performance may be affected by fibrotic overgrowth, host immune responses to alginate, mechanical fragility, and limited proliferation of encapsulated cells (Kavand et al., 2024). To date alginate microcapsules have not been used to deliver Klotho expressing cells.

Chitosan and gelatin-based microcapsules provide attractive biological properties, including biocompatibility, biodegradability, and bioactivity, but their relatively rapid degradation, lower mechanical robustness, and less established long-term implantation record may limit their suitability for sustained therapeutic protein production (Haider et al., 2024; Zhai et al., 2023). Agarose microcapsules offer improved mechanical stability and resistance to enzymatic degradation, allowing long-term containment of transplanted cells; however, limited cell adhesion, potential mass-transfer constraints in larger capsules, and a comparatively modest clinical history remain important considerations (Dutcher et al., 2023). Again, chitosan, gelatin and agarose encapsulation has not yet been tested in conjunction with Klotho expressing cells.

Among the cell-encapsulation approaches reviewed, cellulose sulphate microcapsules (Cell-in-a-Box®) combine several characteristics that are particularly advantageous for long-term Klotho replacement therapy. Unlike alginate, chitosan, gelatin and agarose based systems in which encapsulated cells remain largely static, with the matrix performing more like a “sponge”, cells can proliferate within the cellulose sulphate matrix which has a viscous liquid centre, enabling robust and reproducible capsule loading while maintaining long-term viability and continuous protein secretion. The capsules provide effective immune-isolation, good mechanical stability, and reliable confinement of the encapsulated cell population, while also supporting centralized manufacturing through long term cryopreservation and frozen distribution (Salmons et al., 2010). Although protein output remains dependent on cell viability and local vascularization, these advantages collectively suggest that cellulose sulphate microcapsules may potentially offer a superior balance of durability, manufacturability, immune-protection, and sustained protein production compared with alternative delivery platforms, making them particularly attractive for chronic Klotho replacement strategies.

Candidate cells for such sustained protein production include MSCs as described for the Lionheart KlothoPatch™ II (Lionheartlongevity, 2026) and stable immortal cell lines. Klotho overexpressing MSCs were used in the PLGA/SilMA hydrogels (Sun et al., 2024) and have been used to generate Klotho-enriched extracellular vesicles (EV) which have been shown to be bioactive after injection into a murine model of acute kidney injury generated by glycerol injection (Grange et al., 2020) as well as in a rhabdomyolysis induced acute kidney injury model (Deng et al., 2025).

Klothonova, a joint venture between Austrianova and Avaí Bio, is investigating the use of Cell-in-a-Box® porous cellulose beads (Salmons and Gunzburg, 2023) for the encapsulation of immortal human cells engineered to stably produce soluble Klotho. This encapsulation system is designed to enable continuous secretion of Klotho while providing immune isolation, as the capsule permits diffusion of proteins and small molecules but restricts direct immune cell access. Evidence from prior studies not involving Klotho production suggests that encapsulated cells can remain viable for extended periods. For example, Cell-in-a-Box encapsulated cells have been reported to be present for up to 2 years in human pancreatic cancer trials (Löhr et al., 2014). Intact capsules containing viable cells have also been retrieved months after implantation in dogs to treat mammary cancer (Michałowska et al., 2014) as well as in rats for the treatment of diabetes (Salmons et al., 2014), and in mice for the long term production of monoclonal antibodies (Dangerfield et al., 2013) or in a treatment focused on solid tumors (Salmons and Gunzburg, 2023). These findings suggest that Cell-in-a-Box capsules can support long-term survival of encapsulated cells under a range of implantation conditions, including xenogeneic transplantation settings. Furthermore, the encapsulation system has undergone clinical evaluation in pancreatic cancer patients, albeit to deliver a drug activating system and not Klotho, in two clinical trials that took place in Germany, where safety and feasibility were demonstrated (Löhr et al., 2014). Klothonova has now generated Cell-in-a-Box encapsulated cells showing long term Klotho production and secretion out of the capsules *in vitro* (WHG personal communication).

While these findings provide a foundation for exploring Klotho-based therapies, several limitations should be acknowledged. The majority of the Cell-in-a-Box related clinical data derive from oncology applications rather than regenerative or metabolic indications, and long-term persistence and secretion dynamics in humans remain incompletely characterized. Interspecies differences in immune response and metabolism may limit direct extrapolation of animal data to clinical practice, although use of encapsulated human cells in humans should minimize such effects. Additionally, although encapsulation offers immune protection, the possibility of local inflammatory reactions, device malfunction, or variability in secretion rates cannot be excluded. Thus, it would be advisable to conduct first in man trials with such Klotho-secreting cells using a removal implant. Such studies should also examine the effects of different doses which can be manipulated by choosing a suitable level cell clone and/or by changing the number of capsules that are implanted.

Taken together the encapsulation platform warrants further investigation as a strategy for sustained protein delivery with immune isolation. However, further clinical studies specifically for the use of this technology to deliver Klotho are required to establish the dosing, durability, safety, and therapeutic relevance of Klotho secretion in human populations.

## 5 Discussion

Encapsulated Klotho therapy represents a novel approach that may address some limitations of existing Klotho delivery strategies for age-related diseases by maintaining steady physiological levels of this longevity protein. By shielding human allogenic Klotho-producing cells within immune-protected capsules, it is intended to provide sustained secretion that could potentially mitigate pathological processes associated with neurodegenerative, renal, and cardiovascular disorders (see Figure 2), assuming the correct dosage can be found. Extending beyond single-organ benefits, sustained systemic Klotho exposure may influence multiple biological pathways associated with aging, potentially “refurbishing” the body’s molecular fabric, although the clinical relevance of these effects remains to be established.

**FIGURE 2:**
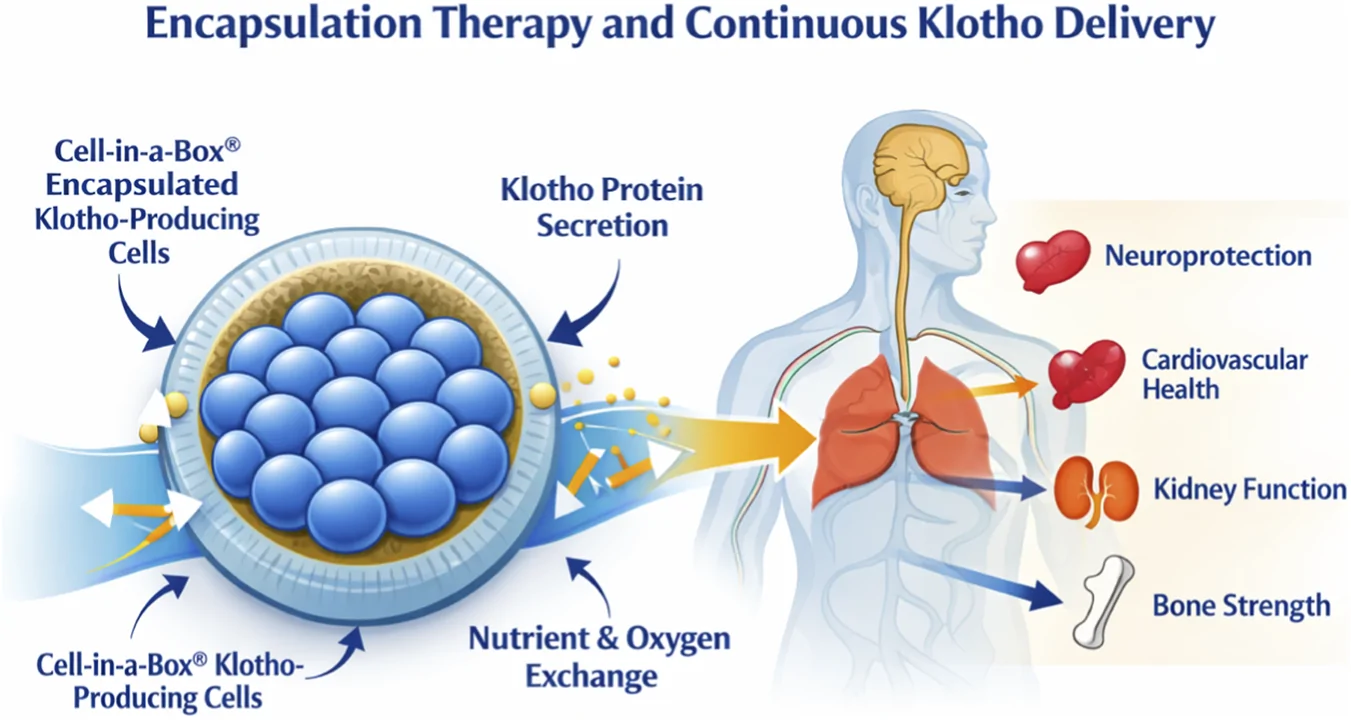
Potential encapsulation therapy using Cell-in-a-Box®. Schematic representation of Cell-in-a-Box® capsules containing Klotho-producing cells. The porous cellulose membrane allows bidirectional exchange of nutrients and oxygen while enabling continuous secretion of soluble Klotho protein. This encapsulation protects cells from immune attack and supports long-term viability. The right of the illustration depicts the systemic benefits from sustained Klotho release. Arrows indicate diffusion of Klotho from encapsulated cells toward major target organs, promoting neuroprotection, cardiovascular health, kidney function, and bone strength. The concept demonstrates how encapsulated cell therapy can maintain physiological Klotho levels.

Emerging research continues to reveal Klotho’s intricate interactions with Wnt signaling, TGF-β pathways (Marwein et al., 2025) and oxidative stress regulators (Prud’homme and Wang, 2026) underscoring its role as a central node in cellular homeostasis. These insights suggest that combining encapsulated Klotho delivery with stem-cell or metabolic therapies could amplify regenerative outcomes. Meanwhile, interest in therapeutic Klotho restoration has increased in recent years, as reflected by multiple emerging development programs, with initiatives such as Klothea Bio’s mRNA therapeutic (AKL003) and Minicircle’s nonviral gene therapy expanding the clinical landscape. Against this backdrop, the Klothonova approach combines an encapsulation technology that has previously undergone clinical evaluation in other indications with the possibility of sustained Klotho secretion from encapsulated cells, capable of sustained physiological benefit and may address some of the limitations associated with transient protein administration and gene-transfer approaches.

Despite considerable advances in understanding Klotho biology, effective long-term delivery of therapeutically relevant concentrations of Klotho remains a significant translational challenge. Encapsulation-based approaches may provide a means of achieving sustained protein exposure while maintaining cell confinement and immune isolation. In contrast to repeated administration of recombinant protein or gene-transfer strategies that are difficult to reverse once administered, a removable, encapsulated cell therapy could potentially offer adjustable long term Klotho production. However, substantial preclinical and clinical work remains necessary to define the optimal dose, duration of expression, long-term safety profile, and therapeutic benefit of sustained Klotho delivery in human disease. Whether such approaches can translate the broad biological activities of Klotho into clinically meaningful outcomes remains an important question for future investigation.

## Data Availability

The original contributions presented in the study are included in the article/supplementary material, further inquiries can be directed to the corresponding author.
